# Evidence of natural selection and dominance of SARS-CoV-2 variant Lambda (C.37) over variants of concern in Cusco, Peru

**DOI:** 10.1007/s00705-022-05645-x

**Published:** 2023-02-14

**Authors:** Maria Antonieta Quispe-Ricalde, Hugo G. Castelán-Sánchez, Pablo M. Meza-Rodríguez, Sonia Dávila-Ramos, José Luis Sierra, Ramón Batista-Garcia, Fátima Concha-Velasco, Sonia Flores Lucana, José De Santa Cruz, Víctor Zea, Marco Galarza, Omar Caceres-Rey, Pablo Tsukayama, Pilar Foronda, Brandon Jason Soto-Chambi, Nestor Abreu

**Affiliations:** 1 Departamento de Biología, Facultad de Ciencias, Universidad Nacional de San Antonio Abad del Cusco, Av. de La Cultura 733, C.P. 0800 Cusco, Perú; 2 Programa de Investigadoras e Investigadores por México. Grupo de Genómica y Dinámica Evolutiva de Microorganismos Emergentes, Consejo Nacional de Ciencia y Tecnología, Av. Insurgentes Sur 1582, Crédito Constructo, Benito Juárez, Ciudad de México, C.P. 03940 México; 3 Centro de Investigación en Dinámica Celular, Instituto de Investigaciones en Ciencias Básicas y Aplicadas, Universidad Autónoma del Estado de Morelos, Morelos. Av. Universidad 1001. Col. Chamilpa, Cuernavaca, Morelos C.P. 62209 México; 4 Escuela de Postgrado, Universidad Nacional de San Antonio Abad del Cusco, Av. de La Cultura 733, Cusco, C.P. 0800 Perú; 5 Laboratorio Regional de Referencia, Gerencia Regional de Salud Cusco, Av. de La Cultura 147, Cusco, C.P. 08003 Perú; 6 Dirección de epidemiología e investigación. Gerencia regional de salud, Av. de La Cultura 147, Cusco, C.P. 08003 Perú; 7 Laboratorio de Referencia Nacional de Biotecnología y Biología Molecular, Instituto Nacional de Salud, Lima, Perú; 8 Laboratorio de Genómica Microbiana, Universidad Peruana Cayetano Heredia, Av. Honorio Delgado 430, San Martín de Porres 15102, Lima, C.P. 15102 Perú; 9 Instituto de Medicina Tropical Alexander von Humboldt, Av. Honorio Delgado 430, San Martín de Porres, Lima, Peru; 10 Wellcome Sanger Institute, Saffron Walden, Cambridge, Reino Unido. Z.P. CB10 1SA, Hinxton, UK; 11 University Institute of Tropical Diseases and Public Health of the Canary Islands, Av. Astrofísico FranciscoSánchez, s/n, San Cristóbal de La Laguna, Tenerife C.P.38200 Spain; 12 NERTALAB, SL., C, /José Rodríguez Mouré, 4, Santa Cruz de Tenerife, Tenerife C.P. 38008 España

## Abstract

Severe acute respiratory syndrome coronavirus 2 (SARS-CoV-2) lineage C.37 (Lambda) has spread rapidly in Peru and other Latin American countries. However, most studies in Peru have focused on Lima, the capital city, without knowing the dynamics of the spread of the variant in other departments. Cusco, Peru, is one of the most popular departments in the country for tourists, so the introduction of new variants of SARS-CoV-2 might occur despite closure of the borders. Therefore, in this work, we analyzed the variants circulating in Cusco. The aim of this work was to better understand the distribution of SARS-CoV-2 lineages circulating in Cusco and to characterize the genomes of these strains. To this end, 46 SARS-CoV-2 genomes from vaccinated and unvaccinated patients were sequenced in the first half of 2021. The genomes were analyzed using phylogenetic and natural selection methods. Phylogenetic trees from Cusco showed dominance of the Lambda lineage over the variants of concern (VOCs), and there was no clustering of variants by district. Natural selection analysis revealed mutations, mainly in the spike protein, at positions 75, 246, 247, 707, 769, and 1020. In addition, we found that unvaccinated patients accumulated more new mutations than did vaccinated patients, and these included the F101Y mutation in ORF7a, E419A in NSP3, a deletion in S (21,618-22,501), and a deletion in ORF3a (25,437-26,122).

**Supplementary Information:**

The online version contains supplementary material available at 10.1007/s00705-022-05645-x.

## Introduction

On December 31, 2019, the novel coronavirus SARS-CoV-2, which causes the disease COVID-19, was first reported in Wuhan, China [1]. From that date, the virus began to spread worldwide. SARS-CoV-2 has a positive-sense single-stranded RNA genome of approximately 29 kb that is responsible for severe acute respiratory illness in humans [2]. The genome of SARS-CoV-2 encodes the structural proteins spike (S), envelope (E), membrane (M), and nucleocapsid (N), and ORF1ab (~21.291 nt) encodes 16 non-structural proteins: leader protein, nsp2, nsp3, nsp4, 3C-like proteinase, nsp6, nsp7, nsp8, nsp9, nsp10, RNA-dependent RNA polymerase, helicase, 3'-5' exonuclease, endoRNase, 2'-O-ribose methyltransferase, and nsp11 [2, 3].

The virus quickly spread worldwide, and variants then began to appear. To date, there are 1479 variants according to the Pangolin classification [4], which are classified into variants of concern (VOC) and variants of interest (VOI) according to the World Health Organization (WHO) (https://www.who.int/es/activities/tracking-SARS-CoV-2-variants).

In 2021, 197 million cases and more than 4.7 million deaths were reported in the world population. In Peru, there have been 1.8 million positive symptomatic cases of COVID-19 and 196,000 deaths [5] (https://covid19.minsa.gob.pe/sala_situacional.asp).

Mortality and infection rates are high, and whole-genome sequencing is needed to follow the evolution and epidemiology of the virus in Peru. Currently, 4,079 virus sequences from Peru have been deposited with the Global Initiative on Sharing Avian Influenza Data (GISAID). By sequencing the complete genome, it is possible to track the dynamics and epidemiology of the virus. For example, in April last year, the predominant lineages were B.1 and B.1.1 [5, 6]. However, recently, the situation changed, as it has been shown that the Lambda variant (C.37) is generally predominant in Peru [5]. In 2021, 74,152 cases were reported in Cusco in early October, with a lethality of 3.94%, and Lambda was the predominant variant, followed by Gamma (P.1) and Delta (B.I.617.2) (https://web.ins.gob.pe/es/covid19/secuenciamiento-sars-cov2).

Despite sequencing efforts, very few sequences have been deposited in the GISAID database. Therefore, in this work, we performed genomic surveillance of SARS-CoV-2 in the Cusco region in the first half of 2021, as this region has a high level of tourist activity, making it more likely for VOC introductions may occur. However, the Alpha and Gamma variants did not predominate over the Lambda variant in the studied patients in the Cusco region.

In this work, the genome sequences of 46 SARS-CoV-2 isolates from vaccinated and unvaccinated patients were determined in the first half of 2021. The genomes were analyzed using phylogenetic methods, and natural selection was evaluated. A high prevalence of the Lambda lineage was observed in the Cusco region, whereas VOC variants were not prevalent. Moreover, unvaccinated patients were more likely to accumulate new mutations than vaccinated patients.

## Materials and methods

### Ethical approval

This study was reviewed by the Institutional Ethics Committee of the Universidad San Antonio Abad del Cusco (UNSAAC) and approved under the number CBI-UNSAAC2021-01.

### Sample collection

Nasopharyngeal swabs were collected from 47 patients in a volume of 2 ml of viral transport medium (VTM), RNA extraction was performed using a Maxwell RSC Viral RNA Extraction Kit (Promega), and the diagnosis of COVID-19 was made based on RT-qPCR using a Novel Coronavirus (2019-nCoV) Nucleic Acid Diagnostic Kit (Sandure Biotech). Samples with a C_t_ value of 25 or below were used for sequencing.

### Whole-genome sequencing

Whole-genome sequencing (WGS) of SARS-CoV-2 isolates (n = 47) was performed on a MiSeq sequencing platform (Illumina, San Diego, CA, USA) with a paired-end read configuration (2 × 150 bp reads) at the Instituto Nacional de Salud de Peru (NIH-Peru) using the CleanPlex® SARS-CoV-2 panel (Paragon Genomics) by amplicon-based target enrichment.

### Viral genome assembly

Reads were verified by quality control using FASTQC v0.11.9 software [7] and then trimmed using Trimmomatic v0.39 [8]. Reads were assembled by mapping to the Wuhan-Hu-1 reference sequence (MN908947) using Bowtie2 v2.3.4.3 [9], and the sam file was converted to fastq using samtools v1.3 [10]. Reads were used for *de novo* assembly using SPAdes v3.14.0 [11], and consensus genome sequences were constructed from contiguous sequences using CONTIGuator [12]. The ancestry of the genomes was determined according to the Pango lineage system, using Pangolin v2.2.2 [13].

### Phylogenetic analysis

Full-length genome sequences of SARS-CoV-2 isolates collected in Peru between February 1, 2021 and August 1, 2021 [14], including sequences from Cusco, were aligned using MAFFT v7.1. The 47 viral consensus genome sequences from Cusco were used to construct a maximum-likelihood (ML) phylogenetic tree using IQ-treev [15] with 1000 ultrafast bootstrap replicates.

### Identification of mutations

Synonymous and non-synonymous substitutions unique to the Cusco sequences were identified using CoV-GLUE [16]. A heat map of mutation frequency was generated using ggplot2 in R.

### Natural selection inference analysis

First, ORFs were identified using the RCoV19 Resource for Coronavirus 2019 NGDC online software [17]. To identify sites subject to pervasive diversifying or purifying selection, we used FEL (fixed-effects likelihood) [18], SLAC (single-likelihood ancestor count) [18], and FUBAR (fast unbiased Bayesian approximation) [19]. To evaluate pervasive and episodic diversifying selection, the mixed-effects model of evolution (MEME) [20] was used.

### Protein structure prediction

The viral proteins listed in Table 1 were modeled using the I-TASSER server [21]. The generated models were visualized in the UCSF program Chimera-alpha [22] by comparing MatchMaker structures using the Needleman-Wunsch alignment algorithm and the BLOSUM-62 matrix to perform sequence alignment from subsequent structural matches and to identify the selected sites.

**Table 1:** Episodic and pervasive natural selection in ORFs of SARS-CoV-2 analyzed using MEME, FEL, SLAC, and FUBAR

ORF	Mixed-effects model of evolution (MEME) p-value threshold of 0.1	Fixed-effects likelihood (FEL), *p*-value threshold of 0.1	Single-likelihood ancestor counting (SLAC), *p*-value threshold of 0.1	Fast unconstrained Bayesian approximation (FUBAR), posterior probability of 0.9
ORF1ab helicase	1113, 2097	0	0	555, 559, 580
ORF1ab nsp2	26, 42, 186, 196, 223, 248, 263, 265, 274, 289	0	0	0
ORF1ab nsp3	62, 126, 148, 196, 204, 1004, 1215, 1243, 1279, 1282, 1287, 1314, 1328, 1365, 1427, 1504, 1589, 1690	62	0	62
ORF1ab nsp4	0	0	0	264
ORF1ab nsp6	0	0	0	4
N	2, 202, 333, 379	2, 202, 366	0	2, 13, 80, 202, 327, 333, 366
ORF3a protein	172	0	0	66
ORF8 protein	35, 36, 121	121	0	121
Surface glycoprotein	5, 12, 18, 246, 247, 250, 385, 515, 769	0	0	75, 246, 247, 707, 769, 1020

## Results and discussion

### Samples

We selected 46 nasopharyngeal swab samples from COVID-19 patients in the Cusco region with a cycle threshold (C_t_) value of 15 for detection of SARS-CoV-2 by qRT-PCR. In total, there were 30 women and 14 men, with a mean age of 37.4 years, 21 of whom were vaccinated and 25 of whom were unvaccinated. All patients had symptoms such as cough, fever, body aches, headache, and anosmia, and none of them died (Supplementary Table S1).

### Genomic diversity of SARS-CoV-2 in Cusco, Peru

In Peru, a major effort has been made to sequence the genomes of SARS-CoV-2 strains circulating in the country. As part of the Genomic Surveillance Network, the Universidad Nacional de San Antonio Abad del Cusco (UNSAAC) and the Universidad Peruana Cayetano Heredia (UPCH) sequenced 47 SARS-CoV-2 genomes from the first half of 2021 to analyze the distribution of circulating lineages in the department of Cusco.

In addition, 3,545 SARS-CoV-2 genomes with higher coverage from Peru were analyzed during the same period to compare the dynamics of SARS-CoV-2 at the national level and in the department of Cusco.

**Fig. 1:**
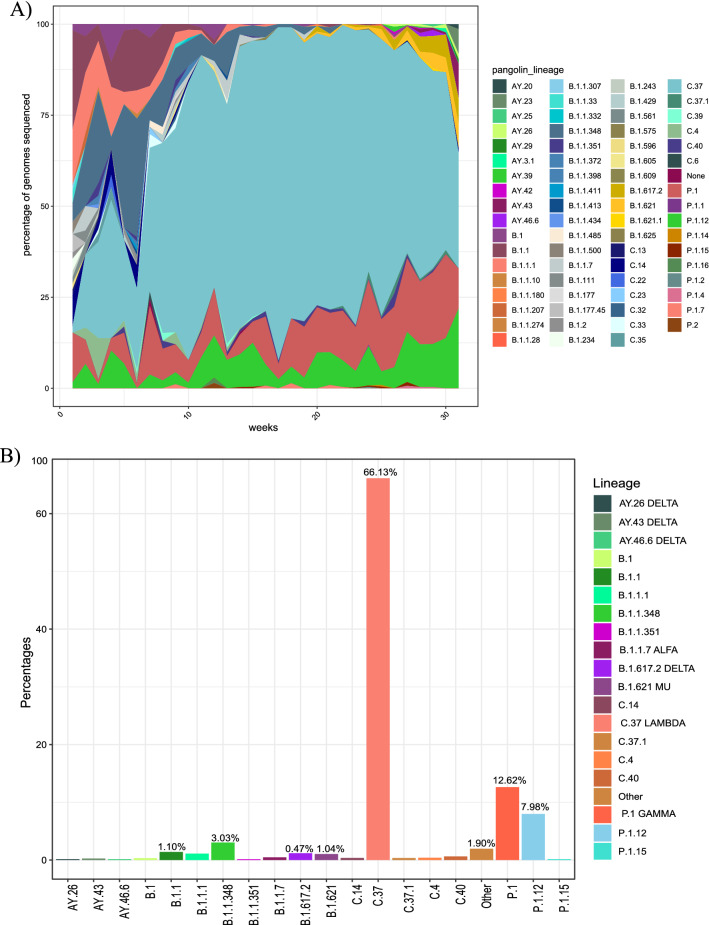
Lineages Circulating lineages in Peru during 2021. Distribution of lineages in Peru between January 2021 to August 2021. (A) Most frequent lineages in Peru. (B) Temporal distribution of lineages in Cusco between January 2021 and August 2021

At the national level, 71 different lineages were found, with the dominant lineage being C.37 (66.14%), followed by P.1 (12.62%), P.1.12 (7.96%), B.1.1.348 (3.03%), and B.1.1 (1.10%). According to the World Health Organization (WHO), the Lambda variant (C37/ GR /452Q.V1/21G) is classified as a VOI (Fig. 1a). In Peru, Lambda was the variant that dominated the first half of 2021; it began to increase in March and declined slightly during the week of June 30 (Fig. 1B). The first sequenced samples of Lambda were reported in Peru in August 2020 but spread rapidly throughout South America, in Chile, Argentina, Ecuador, Colombia, and Brazil [6].

The Lambda variant (C.37 lineage) contains six substitution mutations (G75V, T76I, L452Q, F490S, D614G, and T859N) and a 7-amino-acid deletion in the N-terminal domain (NTD) (RSYLTPGD246-253N) of the spike (S) protein, as well as six substitution mutations (T1246I, P2287S, F2387V, L3201P, T3255I, G3278S) and a 2-amino-acid deletion (del3675/3677) in ORF1. It also contains the substitution P313L in ORF1b and S84L in ORF8 as well as four substitutions in the nucleoprotein (P13L, R203K, G204R, and G214C).

The mutations in the spike protein are mainly related to greater transmissibility and resistance to antibodies, and this could be a reason for the rapid spread of this variant in the Peruvian population. For example, the T76I mutation increases infectivity, L452Q increases the affinity of the spike protein for ACE2 and contributes to transmissibility, and D253N is also associated with a higher rate of transmissibility. The deletion of seven amino acids in the NTD (RSYLTPGD246-253N) at positions 246-253 results in partial resistance to vaccine immunity [23].

Interestingly, lineages considered to be VOCs were found in a small proportion, such as Alpha (0.37%), Mu (0.37%), and Delta (0.47%), in addition to the Delta sublineages AY.26, AY.43, and AY.46.6 (Fig. 1).

The Gamma lineage P.1 was the second most common, followed by the Gamma sublineage P.1.12. These lineages have mutations in the receptor-binding domain (RDB) of the spike protein that are associated with increased transmissibility. P.1 began to increase in Peru around week 31, but interestingly, the Gamma variant did not replace the Lambda variant (Fig. 1B).

Analysis of the diversity of variants in the Cusco region gave a picture similar to that of the country as a whole, with the Lambda variant predominating. The predominant SARS-CoV-2 variants circulating in Cusco were C.37 (n = 227, 85.66%), P.1 (n = 24, 9.05%), and B.1.1.348 (n = 7, 2.64%), whereas the variants P.1.1.12 (n = 2, 0.75%), B.1.621 (n = 1, 0.37%), C.4 (n = 1, 0.37%), C.40 (n = 1, 0.37%), and B.1.1 (n = 1, 0.37%) were found in a lower proportion. The genomes sequenced in this study were C.37 (90.90%), P.1 (9%), and B.1.1.348 (1%).

VOCs such as Alpha, Gamma, and Delta were found in a low proportion compared to Lambda. VOCs did not predominate in the countryside or in the Cusco region during the peak of Lambda. A similar situation occurred in Mexico, where the Alpha variant was not predominant over the B.1.1.519 variant [24].

The prevalence of Lambda in Peru is probably due to the "founder effect" that occurs when a limited number of individual viruses form a new population during transmission. The viral ancestor C.37 first dominated in the population, after which natural selection and random events increased the frequency of variants, which then became fixed [25].

**Fig. 2:**
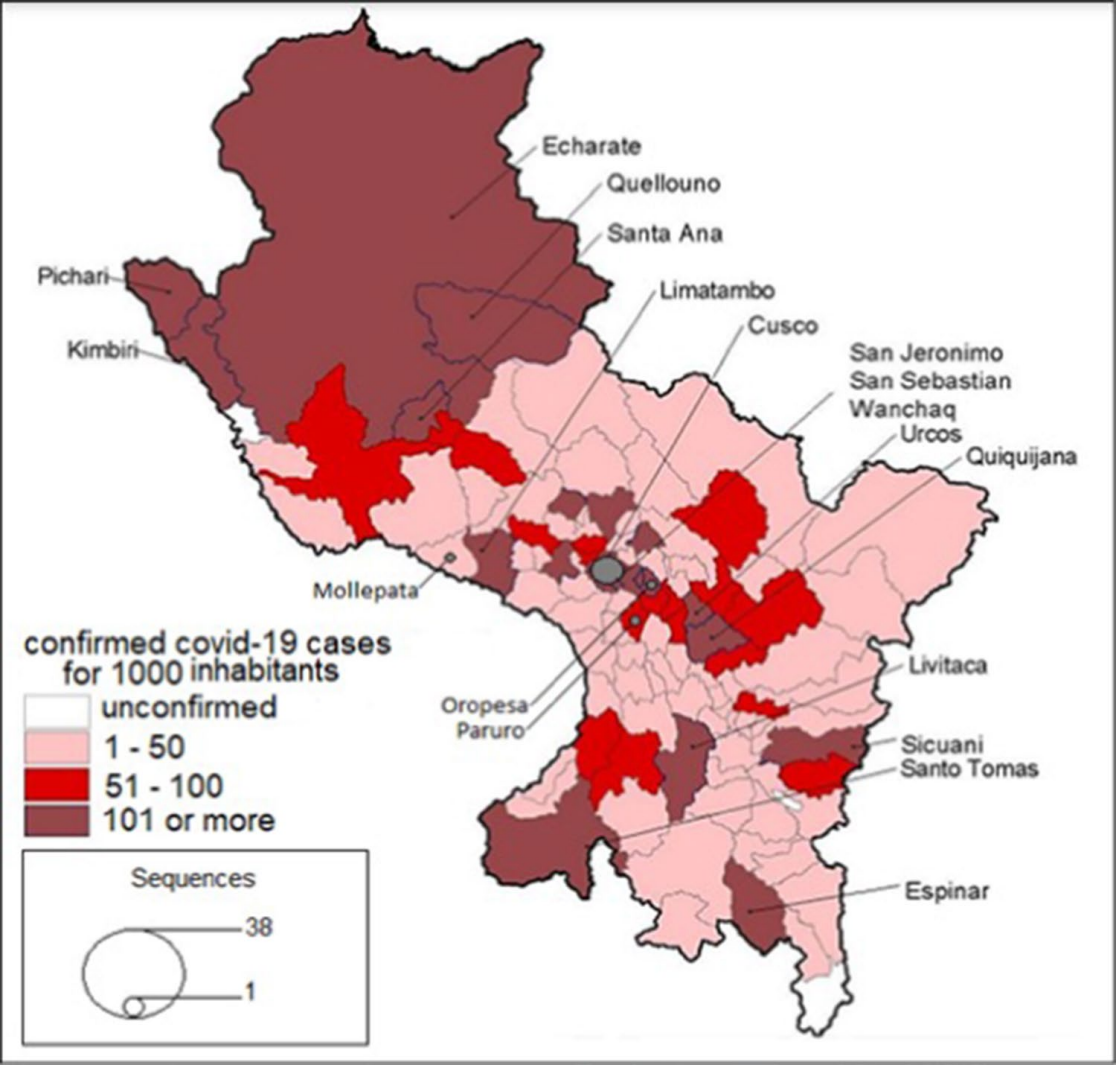
Map of distribution of COVID-19 cases, Cusco. In red, the District of Cusco with most prevail cases

A large proportion of COVID-19 cases in Cusco occurred in the north of the department in the districts of Echarati, Kimbiri, Quellouno, Santa Ana, and Pichari, with more than 100 cases per 1000 inhabitants (Fig. 2). The entire department was affected by an intermediate and high prevalence of COVID-19, with most cases caused by the Lambda lineage.

### Phylogenetic tree of SARS-CoV-2 genome sequences from Peru

**Fig. 3:**
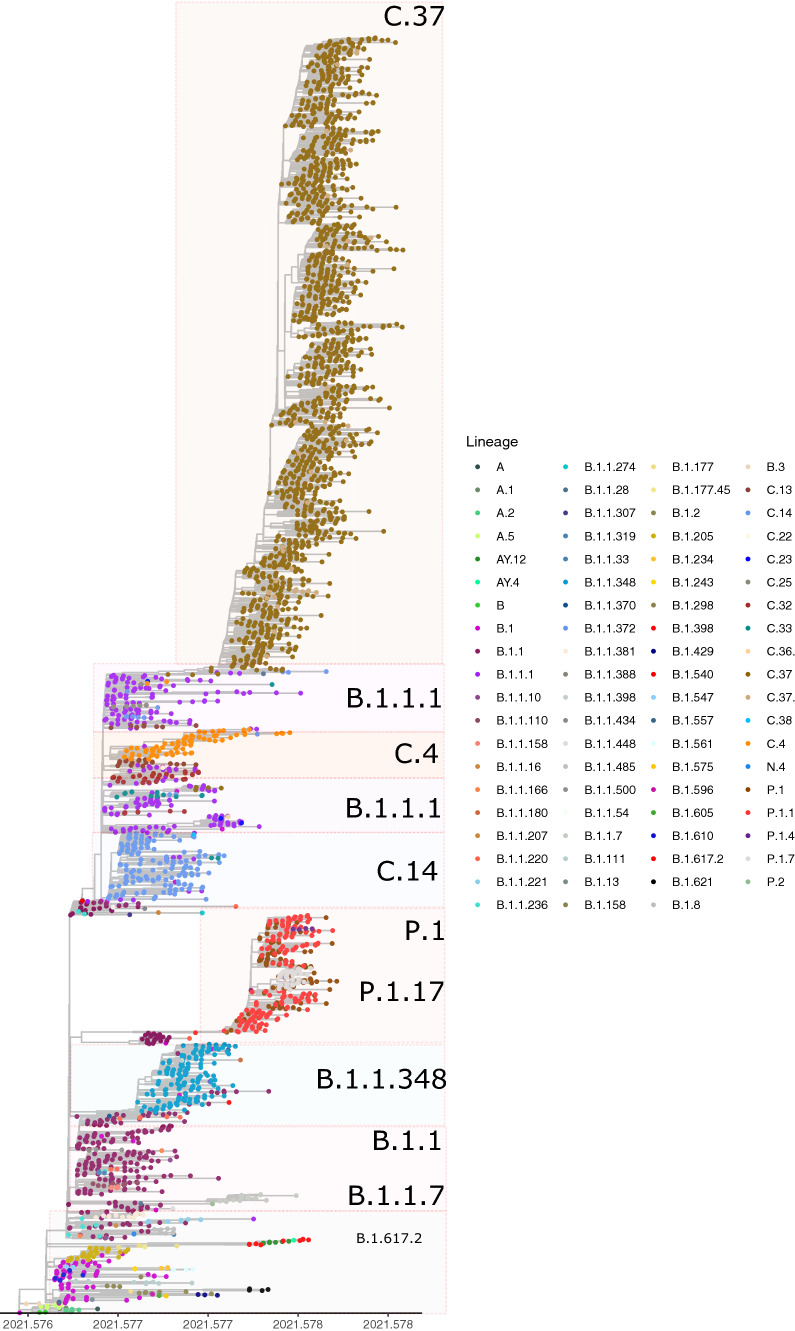
Maximum likelihood phylogeny of lineages from Peru. The tips of the tree are colored by lineage, where C.37 is the most abundant

Phylogenetic reconstruction was performed using 3,545 SARS-CoV-2 genome sequences from Peru that were downloaded from GISIAD, using the sequence from Wuhan, China, to root the tree. Near the root of the tree are the A.1, A.2, and B.2 lineages from China and many global exports to Southeast Asia, Japan, South Korea, Australia, the United States, and Europe (Fig. 3). Thus, these sequences probably represent introductions from other countries.

Each of the dominant lineages in Peru formed a defined clade in the tree. The dominant lineage was C.37, and the Alpha, Mu, Delta, and Gamma VOCs occurred at lower frequencies.

The lineage B.1.1.348 (15%) was predominant in Peru. This lineage is widespread in South America and some North American countries, including Chile (24%), Colombia (8.0%), Ecuador (3.0%), Argentina (3.0%), and the United States of America (38.0%) [26].

Lineage B.1.1.348 has characteristic mutations in the ORF1a (L1175F, V3718F, P314L), S (D614G, R346K, S373P, G1167A), ORF8 (S84L), and N (S2Y, R203K, G204R, R203K) proteins. The R346K mutation in the spike protein is associated with enhanced transmissibility, and S373P is associated with escape from mRNA-vaccine-induced immunity [27, 28]. Possibly due to these mutations, this variant spread rapidly in South America. However, it did not reach the same level of dominance as the C.37 lineage.

Another predominant lineage was B.1.1.1 (Fig. 3). It was predominant in several countries, including the United Kingdom (54.0%), Peru (9.0%), Belgium (4.0%), the United States (3.0%), Italy (2.0%), and Ecuador (1%). Lineage B.1.1.1 is an ancestor of the lineages C.4, C.13, C.14, and C.37, which originated in Peru, and occurred in that country with a frequency of 82.0% to 94.0% (Fig. 3). Later, it spread worldwide, with low percentages found in Europe and North America [29].

The Lambda C.37 lineage was found to be the most common; the predominance of the Lambda variant from January to April may indicate higher transmissibility according to Horizon analysis [5].

Lambda C.37 is a deeply branched sublineage of B.1.1.1 [6], and it was observed with high prevalence in the phylogeny, which may indicate a possible local adaptation. However, Lambda is known to have undergone several different independent introductions, probably from Europe and Asia between mid-February and early March [30] (Fig. 3).

**Fig. 4:**
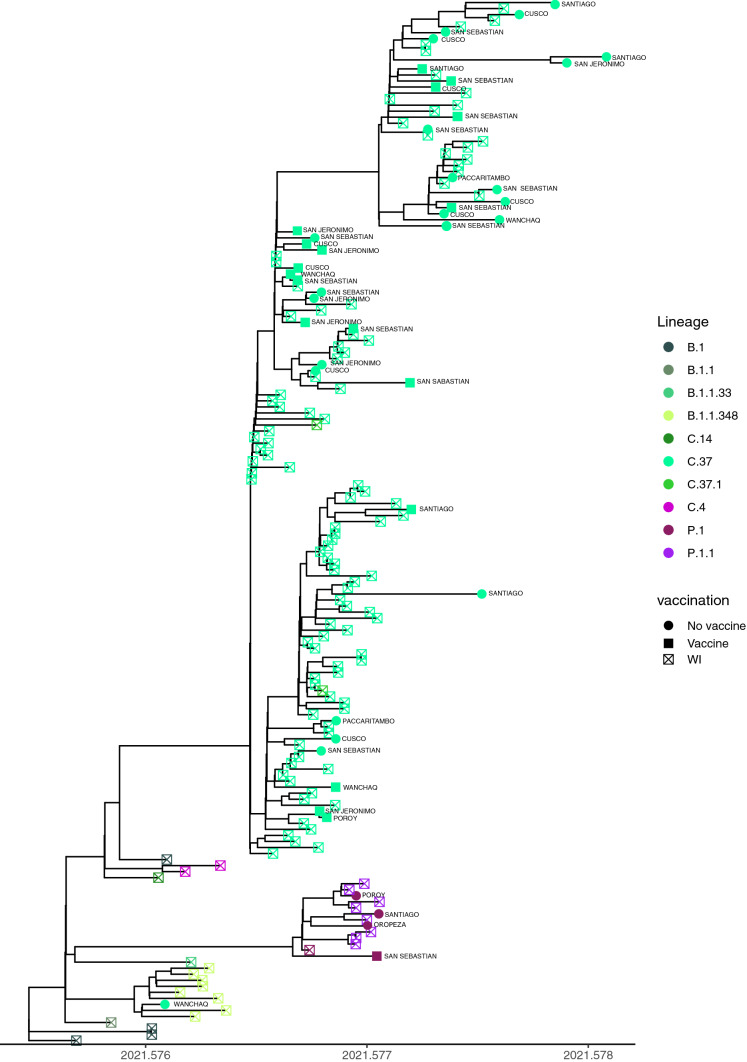
Maximum likelihood phylogeny of lineages from Cusco. The tips of the tree are colored by lineage, additionally in the tips of the tree the localities and vaccination information are observed

The phylogeny based on complete genome sequences from Cusco is shown in Figure 4, with the lineages and the districts from which the isolates were obtained shown at the tips of the tree. A total of 132 complete genome sequences from Cusco were used, 46 of which correspond to the genomes sequenced in this study (Supplementary Table S1). The isolates from Cusco were divided into clades corresponding to lineages C.37 and P.1, and with lineage B.1, which corresponds to imported cases, at the base of the tree.

The Cusco phylogenetic tree did not show any obvious grouping of lineages by district, but there was a grouping by lineage that formed monophyletic clades for each lineage. Lineages B.1 and B.1.1.348 were at the base of the tree. The B.1 lineage was first discovered on February 26, 2020, and it spread worldwide as one of the earliest circulating lineages. The B.1.1.348 lineage was first identified on August 9, 2020. The nationwide prevalence of B.1.1.348 reached 1%, and in Cusco, the B.1.1.348 lineage accounted for 2.64% of the sequences (Fig. 4).

The next clade in the phylogenetic tree of SARS-CoV-2 in Cusco corresponds to the P.1 (Gamma) lineage (Fig. 4). The earliest sequence of the P.1 lineage sampled in Peru was found on December 17, 2020. In Cusco, the earliest sequence was from March 5, 2021. Few sequences are seen in the phylogenetic tree, and in contrast to other regions of the world [31], the Gamma lineage was not predominant in Cusco.

The Cusco isolates belong mainly to the Lambda lineage (C.37) and are distributed in different districts of Cusco; there is no clustering of lineages by province. There was also no obvious clustering of isolates from vaccinated or unvaccinated patients (Fig. 4).

### Mutations and natural selection in SARS-CoV-2 in samples from Cusco

Many of the mutations in the SARS-CoV-2 lineages are characteristic of that lineage and are fixed by natural selection. Natural selection has shaped the evolution of viruses and led to adaptation of viruses to different environments. Because the Lambda variant was dominant in Peru, we investigated the presence of natural selection for specific mutations favoring adaptation in the Cusco region. We use a combination of site-level selection analyses: a mixed-effects evolutionary model (MEME), fixed-effects likelihood (FEL), single-likelihood ancestor counting (SLAC), and fast unbiased Bayesian approximation (FUBAR).

The genes encoding the nonstructural protein NSP3 and the structural proteins S and N, had the highest number of mutations in the samples from Cusco. Regarding the type of selection, some sites were found using the MEME and FUBAR methods to be subjected to episodic selection, especially in the nonstructural proteins (NSPs) (Table 1). No sites were found using SLAC, as this method is the least robust due to its reliance on a relatively naive counting approach [18].

**Fig. 5:**
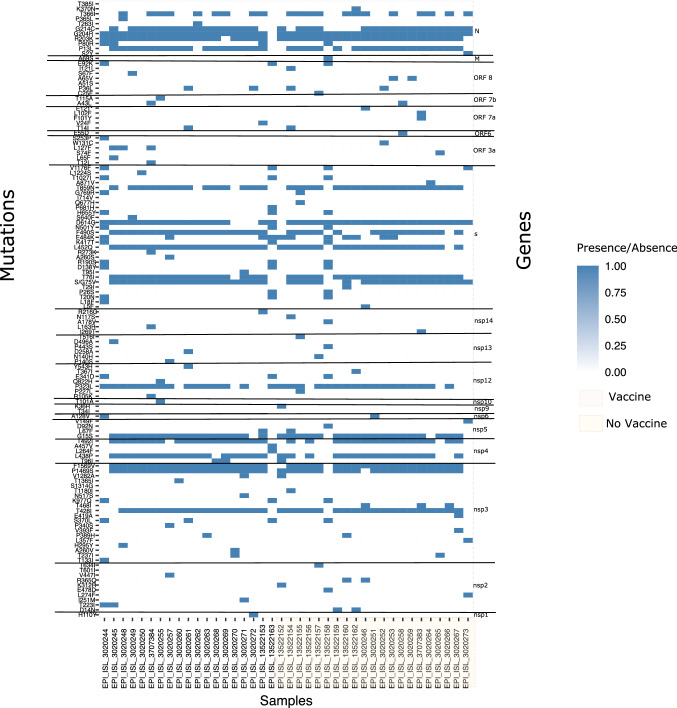
Non-synonymous substitutions in SARS-CoV-2 genome sequences of Cusco compared with the Wuhan reference genome (NC_045512)

Mutations in the NSP2 gene were not identical in samples from vaccinated and unvaccinated patients (Fig. 5). Ten sites in NSP2 were found to be under episodic selection, and the T223I mutation was found in a vaccinated patient. Most of the mutations in NSP2 were found in unvaccinated patients. The NSP2 protein is dispensable for viral replication and can interact with the host proteins prohibitin 1 (PHB1) and prohibitin 2 (PHB2), altering intracellular host signaling [32].

**Fig. 6:**
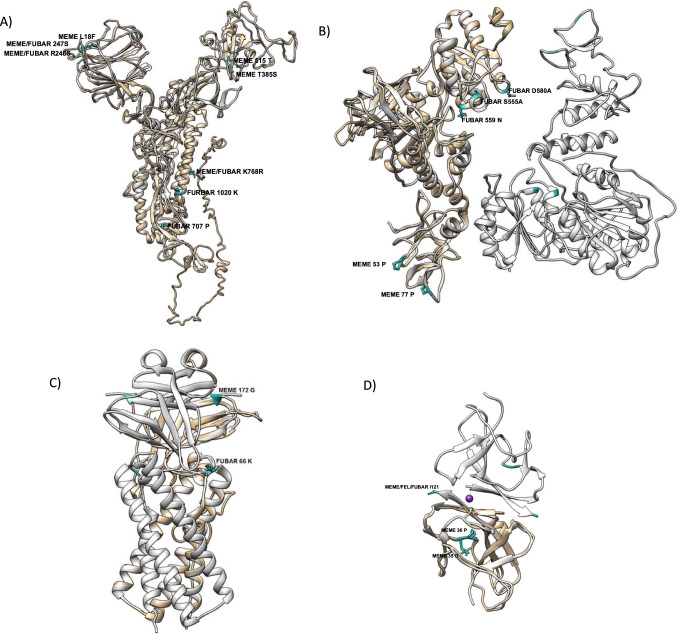
Protein model and sites under natural selection in A) Spike (5X58), B) Helicase (5wwp), C) ORF3a (6xdc), D) ORF8 (7jtl) proteins of SARS-COV-2. In white model and I-TASSER models in tan color. The sites under selection are colored with light sea green accompanied by description of analysis, site and amino acid subject to positive diversifying selection

In the NSP3 protein, the most frequent mutations were T248I, F1469S, and F1569V (Fig. 5). These changes are characteristic of the C.37 lineage and were found in both vaccinated and unvaccinated patients. Analysis of the mode of selection using MEME software revealed that the T1365I mutation is subject to episodic selection and was present only in vaccinated patients (Table 1) (Fig. 6). NSP3 is a transmembrane multidomain protein [33] that is involved in the replication/transcription complex (RTC) and plays a role in polyprotein processing [34]. Interestingly, this protein, which is important for replication, had a greater number of mutations than did non-structural proteins such as NSP1, which is responsible for inhibiting the interferon response at different levels.

The spike gene also accumulated many nonsynonymous mutations. Mutations characteristic of the C.37 lineage (G75V, T76I, L452Q, F490S, D614G, and T859N) were present in both vaccinated and unvaccinated patients. The G75V (under selection) and T76I mutations significantly increase viral infectivity [35]. The mutations L452Q and F490S are responsible for increased transmission [35]. On the other hand, the D614G mutation has been observed in SARS-CoV-2 variants with enhanced viral replication and transmission efficiency. This mutation could be a response to positive selection pressure [36]. The T859N mutation had no effect on vaccine-induced neutralization [35].

The sites under natural selection in the spike protein were analyzed. Several sites were found, with the mutations L18F and G769R being the only ones that exhibited episodic selection in vaccinated patients. The L18F mutation occurred in the South African variant B.1.351 (Beta or GH501Y variant. V2). Later, it was found in the P.1 variant from Brazil (Gamma or GR /501Y variant. V3) and the Zeta variant (P.2). Studies have shown that the L18F substitution can affect neutralizing antibody binding [37].

Some mutations in ORF3a were found to be under episodic selection. ORF3a is known to induce apoptosis, suggesting that ORF3a mutations in SARS-CoV-2 might reduce apoptosis in infected cells, allowing the virus to spread further during infection [38]. Some sites in the ORF3a protein were shown previously to be at under positive natural selection by the FUBAR and DEPS assays, and these might may be under selection from the immune system [3].

Another of the proteins with the highest number of mutations was the structural nucleocapsid phosphoprotein N. The N protein had the characteristic mutations of the C.37 lineage: P37L, R203K, G204R, and G214C. In addition, there was evidence of sites under positive natural selection by the FUBAR method, which had been identified previously by Velazquez-Salinas et al. [3].

In addition, a few nonsynonymous substitutions were observed in ORF8. This protein has been linked to viral pathogenesis by regulating the first innate response to SARS-CoV infection [39]. Previously, it was reported that ORF8-specific sites were under positive selection [3].

Some novel mutations were observed that have not been reported previously. These were present in samples from unvaccinated patients, such as sample CUS-UPCH-0813, with a F101Y mutation in ORF7a (Fig. 5). Sample CUS-UPCH-081 was found to have an E419A mutation in NSP3 and a deletion in S (nt 21,618-22,501). A deletion in ORF3a (nt 25,437-26,122) was also detected in samples CUS-UNSACC-3, 8, 12, and 15 in unvaccinated patients from Cusco. Previous reports have indicated that unvaccinated patients had a higher viral load than vaccinated patients [39], increasing the likelihood of errors in each round of replication. Lower viral replication rates of the Delta and Omicron variants have been observed in vaccinated individuals when compared to unvaccinated individuals [40, 41]. Although vaccination does not confer total immunity to SARS-CoV-2 infection, it does reduce the viral load and thereby the frequency of appearance of new mutations that can become fixed by natural selection.

In general, the NSP3, N, and S proteins in the Cusco samples accumulated a greater number of amino acid substitutions, but this does not mean that they are all under natural selection, because many can be eliminated by purifying selection. The proteins with some sites under positive natural selection were ORF3a, ORF8, and S. Our evolutionary analysis supports the hypothesis that early divergence events during the SARS-CoV-2 pandemic may be associated with positive selection of specific sites in ORF3a and ORF8.

## Conclusions

Our results describe the distribution of SARS-CoV-2 lineages in the department of Cusco and evolutionary events such as natural selection. The Lambda lineage (C37) is predominant in the Cusco region and Peru, followed by the Gamma lineage (P1). The NSP3, S, and N genes accumulated a greater number of nonsynonymous mutations, many of which were not found to be under positive natural selection because they were fixed previously in the C.37 lineage. Isolates from unvaccinated patients had novel mutations not found in those from vaccinated patients.

Sites under directional and episodic selection in viruses from vaccinated and unvaccinated patients were predominantly found in nonstructural proteins, mainly NSP3. However, in the absence of experimental work showing phenotypic differences in SARS-CoV-2 isolates with these mutations, we cannot evaluate the importance of the nonsynonymous changes in viruses from patients from Cusco. Sites under natural selection may have public health implications. We suggest further studies to investigate how these changes affect evolution.

## Supplementary Information

Below is the link to the electronic supplementary material.

Supplementary file1 (XLSX 14 kb)

Supplementary file2 (XLSX 12 kb)

## Data Availability

The data of this study are available in GISAID under the accession number: EPI_ISL_3020244, EPI_ISL_3020245, EPI_ISL_3020248, EPI_ISL_3020249, EPI_ISL_3020250, EPI_ISL_3707384, EPI_ISL_3020255, EPI_ISL_3020257, EPI_ISL_3020260, EPI_ISL_3020261, EPI_ISL_3020262, EPI_ISL_3020263, EPI_ISL_3020268, EPI_ISL_3020269, EPI_ISL_3020270, EPI_ISL_3020271, EPI_ISL_3020272, EPI_ISL_13522153, EPI_ISL_13522163, EPI_ISL_13522152, EPI_ISL_13522154, EPI_ISL_13522155, EPI_ISL_13522156, EPI_ISL_13522157, EPI_ISL_13522158, EPI_ISL_13522159, EPI_ISL_13522160, EPI_ISL_13522162, EPI_ISL_3020246, EPI_ISL_3020251, EPI_ISL_3020252, EPI_ISL_3020253, EPI_ISL_3020258, EPI_ISL_3020259, EPI_ISL_3707383, EPI_ISL_3020264, EPI_ISL_3020265, EPI_ISL_3020266, EPI_ISL_3020267, EPI_ISL_3020273.
